# Enucleation of a Large Pseudo-Mesenteric Cyst with Hemorrhage and Infection: A Case Report

**DOI:** 10.31662/jmaj.2025-0217

**Published:** 2025-09-05

**Authors:** Takeshi Utsunomiya, Jota Watanabe, Ryo Karasudani, Naho Ishimura, Atsushi Takada, Masayuki Kanzaki, Shigehiko Yagi, Hirotsugu Yoshiyama, Satoshi Sumida, Hiromi Ohtani

**Affiliations:** 1 Department of Gastroenterological Surgery, Ehime Prefectural Central Hospital, Ehime, Japan; 2 Department of Pathology, Ehime Prefectural Central Hospital, Ehime, Japan

**Keywords:** pseudo-mesenteric cyst, enucleation, acute abdomen

## Abstract

Pseudo-mesenteric cysts are exceptionally rare lesions, with limited reports of surgical management. This case highlights the novelty of enucleating a large infected pseudo-mesenteric cyst without intestinal resection, emphasizing its clinical significance.

We report the case of a 64-year-old man who presented with acute abdomen. Computed tomography revealed a cystic lesion approximately 17 cm in size with an enhanced capsule in the sigmoid mesentery. Emergency surgery was warranted due to severe inflammation and inferior vena cava compression. Initial laparoscopy ruled out appendiceal abnormalities, and laparotomy was performed. Careful dissection along the thickened cyst wall, as seen on preoperative imaging, enabled complete enucleation without the need for intestinal resection.

This case demonstrates successful enucleation of a giant pseudo-mesenteric cyst, a rare surgical challenge, highlighting the importance of meticulous surgical planning and technique.

## Introduction

Mesenteric cysts are uncommon benign masses, with an incidence of approximately 1 in 20,000 in the pediatric population ^(1)^. Pseudo-mesenteric cysts represent an extremely rare subtype. In terms of location, 60% of cases are reported in the small bowel mesentery, while 24% occur in the large bowel mesentery ^(2)^. We present a case of a large pseudo-mesenteric cyst treated by open enucleation.

## Case Report

**Figure 1.**
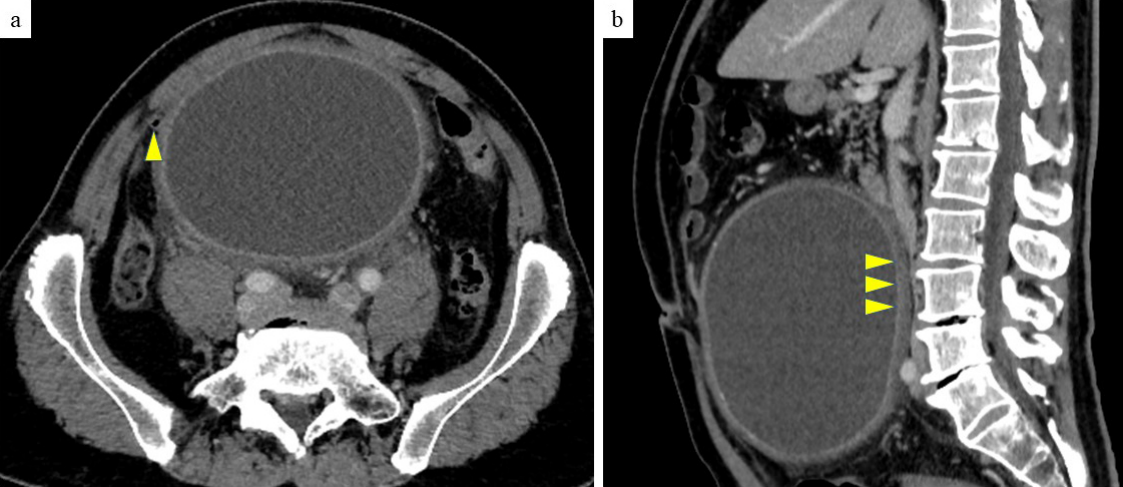
a) Contrast-enhanced horizontal CT image: The appendix is in contact with a large cyst (arrowhead). The continuity between the appendix and cyst is unclear on CT. b) Contrast-enhanced sagittal CT image: The IVC is significantly compressed by the large cyst (arrowhead). CT: computed tomography; IVC: inferior vena cava.

**Figure 2.**
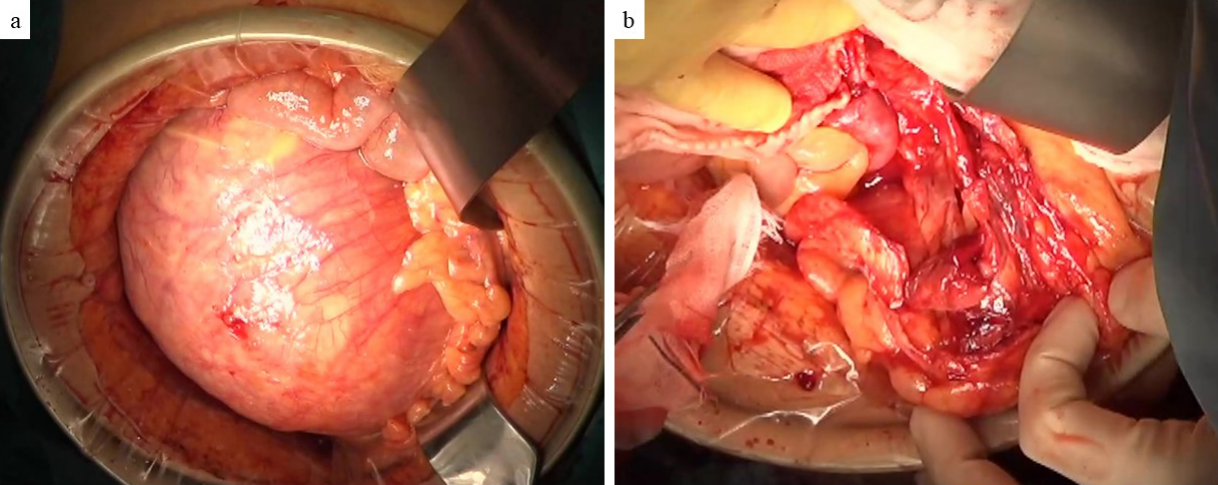
Intraoperative findings. a) The large cyst originated from the sigmoid mesentery. b) Post-enucleation image of the abdominal cavity showing that only the cyst was removed without damage to the ureter or other parts of the colon.

**Figure 3.**
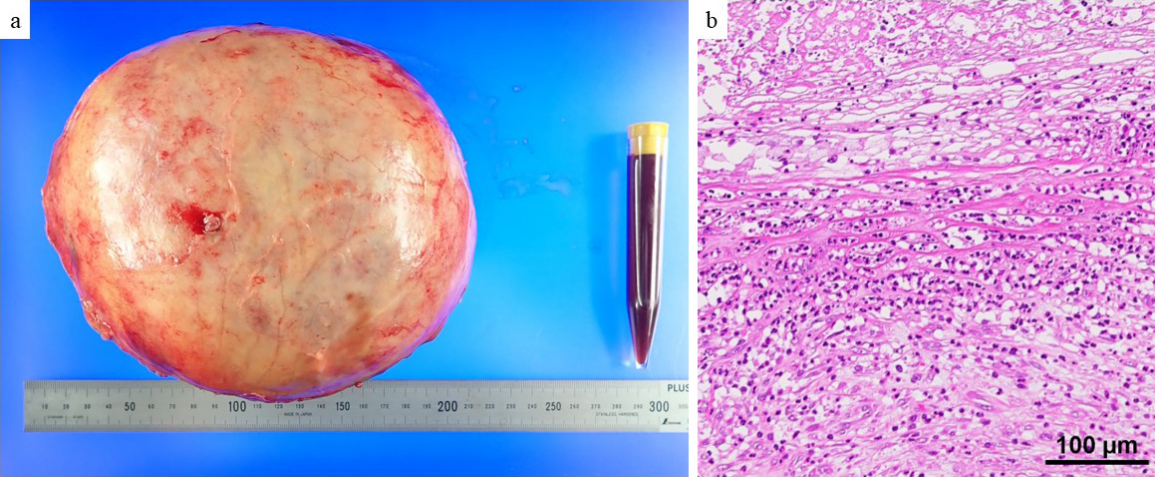
Resected specimen. a) The cyst measured 210 160 mm and was covered by a relatively thick fibrous capsule. The cystic fluid was old bloody. b) Histological image of the cyst wall showing the cystic lumen covered with fibrin, blood, and degenerative material. No epithelial or endothelial cells were present. The cyst wall was composed of fibrous connective tissue with numerous neutrophilic infiltrates.

The patient was a 64-year-old man. Four days before referral to our hospital, he developed right lower abdominal pain, followed by progressive abdominal distention over the next 3 days. He also noticed an increase in his belt hole size during this period. He had no history of abdominal trauma. The patient’s blood biochemical data were as follows: white blood cell count, 9530/μL (3,300-8,600/μL); hemoglobin, 13.2 g/dL (13.7-16.8 g/dL); C-reactive protein, 22.34 mg/dL (0.00-0.14 mg/dL); carcinoembryonic antigen, 2.2 ng/mL (0.0-5.0 ng/mL); carbohydrate antigen 19-9, 8.2 U/mL (0.0-37.0 U/mL); and no hepatic enzymes or renal function abnormalities were noted. Contrast-enhanced computed tomography revealed a cystic lesion approximately 17 cm in length with an enhanced capsule in the sigmoid mesentery. No internal contrast-enhanced nodules were observed, but elevated peri-cystic lipid density suggested inflammation. An infected mesenteric cyst was the most likely diagnosis. The cyst was in contact with the appendix (Figure 1a), raising the possibility of abscess formation due to perforated appendicitis. However, these distinctions were difficult to make with preoperative imaging. The cyst was large, with the superior mesenteric artery and vein draining ventrally, the inferior mesenteric artery draining leftward, and evidence of compression of the inferior vena cava (Figure 1b). Emergency surgery was performed. First, diagnostic laparoscopy was performed to rule out perforated appendicitis. Laparoscopy revealed no evident inflammatory changes in the appendix. Laparotomy was performed. The cyst was located within the mesentery of the sigmoid colon (Figure 2a), but there was no continuity with the intestinal tract. The cyst capsule was relatively thick, allowing for enucleation via sharp dissection along the capsule (Figure 2b). The operation lasted 147 min, with a blood loss of 425 mL. The enucleated mesenteric cyst had a maximum diameter of 21 cm (Figure 3a). The cystic fluid was a light, pleomorphic solution (Figure 3a), with no malignant findings on cytology. The cystic fluid culture tests identified Clostridium ramosum, Eubacterium callanderi, and Parabacteroides distasonis, while no bacteria were detected in the ascitic fluid cultures. Postoperatively, the patient showed overall good progress and was discharged on the 18th postoperative day. Histological examination revealed a monocystic structure with a fibrous connective tissue cyst wall, lacking epithelial or endothelial cells on the luminal surface (Figure 3b). The pathological diagnosis was a pseudo-mesenteric cyst. Eight months post-surgery, no recurrence has been observed.

## Discussion

Infectious pseudo-mesenteric cysts have been reported infrequently, and the mechanism of infection has not been clarified ^(3)^. Some reports cite bacterial translocation as the most likely route of infection ^(3), (4)^. In the present case, there was no obvious connection between the cyst and the intestinal tract, and the bacterial culture of the cystic fluid detected intestinal bacteria, suggesting that intestinal bacteria had invaded the cyst for some reason. The most recent report on pseudo-mesenteric cysts in Japan summarizes cases from 1988 to 2021 ^(5)^. Including other reports and self-experimental cases, a total of 27 cases from 2000 to 2024 are listed in Table 1 ^(6), (7), (8)^. Regarding surgery, laparoscopic surgery was performed in 10 cases. The cyst diameters ranged from 40 mm to 210 mm (median, 100 mm) in laparotomy cases and from 35 mm to 210 mm (median, 47 mm) in laparoscopic cases. Case 13 involved a large cyst with a diameter of 210 mm, but laparoscopic cyst enucleation was performed. Initially, puncture drainage of the cyst was carried out, followed by laparoscopic enucleation ^(9)^. In our case, the cyst was severely inflamed. Severe inflammation makes dissection difficult. There is a risk of massive hemorrhage, so laparotomy was performed. The cyst diameters ranged from 42 mm to 150 mm (median, 105 mm) in patients who underwent complicated bowel resections, while they ranged from 35 mm to 210 mm (median, 55 mm) in patients without complicated bowel resections.

**Table 1.** Summary of Pseudo-Mesenteric Cyst Locations in Japan.

No	Reference	Reported year	Age	Gender	Location	Size (mm)	Trauma history	Laparoscopic operation	Enucleation	Bowel resection
1	(5)	2000	31	Female	Mesojejunum	40	−	−	+	−
2	(5)	2002	66	Male	Mesojejunum	55	−	−	−	+
3	(5)	2003	41	Female	Mesojejunum	40	−	+	+	−
4	(5))	2003	43	Female	Mesotransverse	150	−	−	−	+
5	(5)	2003	38	Female	Mesoileum	110	−	−	−	+
6	(5)	2003	31	Female	Mesosigmoid	100	−	−	−	+
7	(5)	2004	16	Male	Mesojejunum	55	−	−	+	−
8	(5)	2004	74	Male	Mesoileum	60	−	−	−	+
9	(5)	2005	50	Female	Mesoileocecum	140	−	−	−	+
10	(5)	2007	63	Male	Mesojejunum	55	−	+	+	-
11	(5)	2007	84	Female	Mesoileum	65	−	−	−	+
12	(5)	2008	74	Female	Mesojejunum	80	−	−	−	+
13	(5)	2010	76	Male	Ligamentum gastrocolicum	210		+	+	−
14	(5)	2014	65	Male	Mesojejunum	40	−	+	+	−
15	(5)	2014	83	Female	Mesoileocecum	110		−	−	+
16	(5)	2017	64	Male	Mesoaccending colon~mesotransverse colon	120	−	−	−	+
17	(5)	2017	44	Female	Mesojejunum	42	−	+	-	+
18	(6)	2017	47	Female	Meso small intestine	140	−	−	+	−
19	(7)	2018	73	Female	Mesosigmoid	52	−	+	+	−
20	(5)	2018	32	Male	Mesojejunum	55	−	+	+	−
21	(5)	2018	22	Male	Mesojejunum	40	−	+	+	−
22	(5)	2018	60	Male	Meso small intestine	60	−	−	+	−
23	(8)	2019	16	Male	Mesojejunum	65	−	−	+	−
24	(5)	2021	40	Female	Meso ascending colon	142	−	−	−	+
25	(5)	2021	55	Female	Mesojejunum	35	−	+	+	−
26	(5)	2022	33	Female	Mesojejunum	55	−	+	+	−
27	Our case	2024	64	Male	Mesosigmoid	210	−	−	+	−

In our case, the cyst was large enough to consider bowel resection. However, preoperative imaging showed no significant features within the cyst to suggest malignancy, and the cyst wall appeared relatively thick. A decision was made to perform cyst enucleation, which was successfully completed through careful dissection along the cyst wall, without major vascular injury or the need for bowel resection.

## Article Information

### Author Contributions

Takeshi Utsunomiya, Atsushi Takada, and Hirotsugu Yoshiyama performed the surgical procedures. Takeshi Utsunomiya, Ryo Karasudani, Naho Ishimura, Shigehiko Yagi, and Masayuki Kanzaki joined the data interpretation. Takeshi Utsunomiya drafted the manuscript. Jota Watanabe, Hiromi Ohtani, and Satoshi Sumida were involved in key revisions of the manuscript. Hiromi Ohtani joined the final approval of the manuscript. All authors are in agreement regarding the consent of the manuscript. All authors read and approved the final manuscript.

### Conflicts of Interest

None

### Approval by Institutional Review Board (IRB)

Not applicable.

### Informed Consent

The patient authorized the publication of the case while requesting the confidentiality of his identity.
